# Impaired aortic distensibility determined by magnetic resonance imaging in patients with different bicuspid aortic valve phenotypes

**DOI:** 10.1186/1532-429X-11-S1-O71

**Published:** 2009-01-28

**Authors:** Thananya Boonyasirinant, Randolph M Setser, Milind Y Desai, Scott D Flamm

**Affiliations:** Cleveland Clinic, Cleveland, OH USA

**Keywords:** Aortic Aneurysm, Pulse Wave Velocity, Bicuspid Aortic Valve, Marfan Syndrome, Aortic Distensibility

## Introduction

Beyond the morphologic and functional abnormalities of the bicuspid aortic valve (BAV) there is also intrinsic pathology of the aortic wall, manifested by potentially lethal complications such as aortic aneurysm or dissection. Aortic distensibility and compliance are impaired in atherosclerotic aortic aneurysms and Marfan syndrome. Similar abnormalities of compliance are felt to occur in the setting of BAV, though this has been little studied with velocity-encoded magnetic resonance imaging (VENC-MRI), and further there is no data on the influence of BAV morphology on this abnormality. VENC-MRI is a potent non-invasive technique to determine aortic distensibility via aortic pulse wave velocity (PWV) measurements; in addition these measurements do not depend on knowledge of central arterial pressure or geometrical assumptions that may limit alternative measurement tools.

## Purpose

We sought to assess thoracic aortic distensibility by PWV measurements from VENC-MRI in patients with bicuspid aortic valve, and to determine if differences exist between different BAV phenotypes.

## Methods

VENC-MRI was performed in 100 BAV patients and 35 controls (trileaflet aortic valve without dysfunction, and no aortic aneurysm). The PWV was determined between the mid ascending and proximal descending aorta. Velocity measurements were made perpendicular to the long axis of the mid ascending and proximal descending thoracic aorta. The aortic path length between the two locations was directly measured from three-dimensional reconstruction in the oblique sagittal orientation encompassing the aortic arch. The BAV phenotypes were imaged using a cine-SSFP or cine-GRE across the face of the aortic valve, and then classified as: right-left cusp fusion (R-L fusion), right and non-coronary cusp fusion (R-NC fusion), and left and non-coronary cusp fusion (L-NC fusion).

## Results

**Figure 1:**
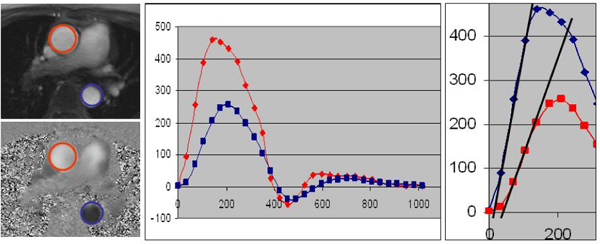
***Left panel*** **: Through-plane velocity-encoding magentic resonance imaging ascending (red circle) and proximal descending aorta (blue circle)**. *Middle panel*: Corresponding flow measurement at ascending (red line) and proximal descending aorta (blue line). *Right panel*: The measurement of arrival time at ascending and proximal descending aorta. The pulse wave velocity was calculated as the aortic path length between these 2 sites divided by the time delay between the arrival of the foot pulse wave at these 2 sites.

**Figure 2:**
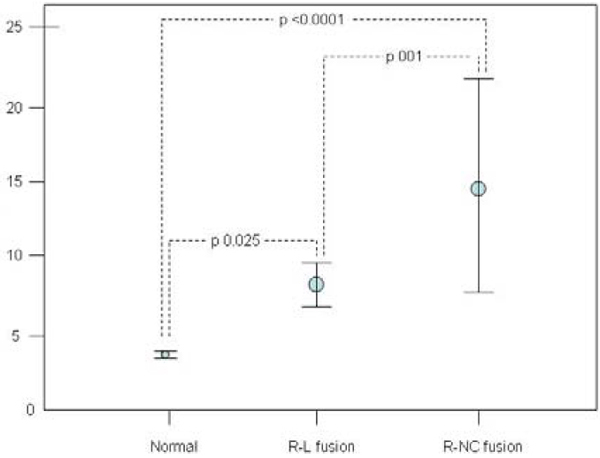
**The pulse wave velocity (PWV) in normal patients, right and left (R-L) fusion, and right and non-coronary (R-NC) bicuspid aortic valve patients**. Circal, mean and whiskers. 95% confidence intervals.

BAV phenotypic classification: 76 R-L, 23 R-NC, and 1 L-NC fusion were identified. Mean age was not significantly different among patients with R-L fusion, R-NC fusion, and controls (49.0, 49.6, and 45.3 years, respectively; p = NS). BAV patients revealed increased PWV compared to controls (9.8 vs. 3.8 m/s; p < 0.0001). Furthermore, PWV was significantly different among patients with R-NC fusion, R-L fusion phenotypes, and controls (14.9, 8.0, and 3.8 m/s, respectively; p < 0.0001). Figures 1 and 2.

## Conclusion

This study, in the largest cohort of BAV patients studied with MRI to date, has identified significantly diminished aortic distensibility (increased PWV from VENC-MRI) compared to controls. Further, this study is the first to demonstrate greater impairment of aortic distensibility in the phenotype of R-NC fusion as compared to R-L fusion. There is a clear association between impaired aortic distensibility and aortic valve configuration; the greater impairment in aortic distensibility in BAV with R-NC fusion phenotype raises concern for amplification of aortic pathology. This differentiation based on valvular phenotype suggests a potentially novel parameter for enhanced surveillance and potentially altered surgical triage in this high risk group.

